# Unrecognized Dengue Transmission in Socially Vulnerable Peri-Urban Neighborhoods of a Temperate Argentine City: Integrating Serology with Knowledge, Attitudes, and Practices

**DOI:** 10.3390/epidemiologia7040099

**Published:** 2026-07-13

**Authors:** Diego A. Mendicino, Tamara Ricardo, Maximiliano A. Cristaldi, Mariana Maglianese, Gastón Guzmán, Sebastián Claussen, Romina Chiaraviglio, Federico Costa, Christian A. Avalos, M. Andrea Previtali

**Affiliations:** 1 Centro de Investigaciones Sobre Endemias Nacionales (CIEN), Facultad de Bioquímica y Ciencias Biológicas (FBCB), Universidad Nacional del Litoral (UNL), Ciudad Universitaria, 3000 Santa Fe, Argentinargchiaraviglio@gmail.com (R.C.); 2 Consejo Nacional de Investigaciones Científicas y Técnicas (CONICET), 3000 Santa Fe, Argentina; christianavalosptp@gmail.com; 3 Departamento de Investigación Epidemiológica, Instituto Nacional de Epidemiología “Dr. J. H. Jara” (INE), ANLIS Malbrán, 7600 Mar del Plata, Argentina; tamararicardo83@gmail.com; 4 Departamento de Ciencias Naturales, Facultad de Humanidades y Ciencias (FHUC), Universidad Nacional del Litoral (UNL), Ciudad Universitaria, 3000 Santa Fe, Argentina; maximilianocristaldi@yahoo.com.ar; 5 Región de Salud Santa Fe, Ministerio de Salud, Gobierno de la Provincia de Santa Fe, 3000 Santa Fe, Argentina; marianadesantafe@yahoo.com.ar; 6 Laboratorio de Epidemiología Provincial de Entre Ríos, 3100 Paraná, Argentina; gastonguzman86@gmail.com; 7 Cátedra de Morfología Normal, Facultad de Bioquímica y Ciencias Biológicas, Universidad Nacional del Litoral (UNL), Ciudad Universitaria, 3000 Santa Fe, Argentina; s.claussen29@gmail.com; 8 Institute of Collective Health, Federal University of Bahia, 40110-040 Salvador, Brazil; fcosta2001@gmail.com; 9 Instituto Gonçalo Moniz, Fundação Oswaldo Cruz, Ministério da Saúde, 40296-710 Salvador, Brazil

**Keywords:** dengue, serological analysis, asymptomatic cases, risk factors, viral zoonotic disease, epidemiology

## Abstract

**Background/Objectives:** Dengue is an emerging arboviral disease in temperate South America, where urban expansion, climate variability, and social vulnerability favor transmission. In Argentina, the endemic circulation of dengue was established in the late 1990s and outbreaks are reported every three or four years. **Methods:** This cross-sectional study assessed dengue virus (DENV) seropositivity and associated sociodemographic, environmental, and knowledge-attitudes-practices (KAPs) factors in three socioeconomically vulnerable peripheral neighborhoods of Santa Fe, Argentina, between December 2019 and March 2020. **Results:** A total of 188 adults were surveyed and tested for anti-DENV IgG using ELISA. KAPs questionnaires and direct peridomiciliary observations were used to characterize exposure contexts. Apparent seropositivity was 16.5%, with an adjusted estimate of 10.7% after accounting for test performance, indicating substantial unrecognized DENV circulation. Most seropositive individuals had no previous dengue diagnosis, highlighting underdetection likely related to asymptomatic infections, limited healthcare access, or surveillance gaps. Although dengue awareness was high (98.4%), knowledge was often incomplete and was weakly correlated with preventive practices, suggesting that awareness alone does not translate into effective risk reduction under structural constraints. In multivariate analysis, living farther from vacant lots was associated with higher odds of seropositivity, consistent with transmission concentrated in denser urban settings. **Conclusions:** Integrating serology with KAPs surveys provides critical insights into hidden transmission and supports targeted surveillance and public health interventions in vulnerable urban settings.

## 1. Introduction

The emergence and re-emergence of infectious diseases (EID) have been increasingly linked to climate change, raising significant public health concern, particularly in the aftermath of the COVID-19 pandemic [1,2]. An EID that is spreading in urban areas of subtropical and temperate regions is Dengue fever, which is caused by dengue virus (DENV serotypes 1–4) and transmitted by *Aedes aegypti* and *Aedes albopictus* mosquitoes [3,4].

*Aedes* mosquitoes have adapted to breed in water containers found in and around homes, with dengue cases peaking during warm and humid months [3,4]. In Argentina, dengue re-emerged in 1998, with periodic outbreaks in the northern region since then [5,6]. Despite being a notifiable disease, dengue remains underreported due to asymptomatic cases or misdiagnosis as other infections [4,7]. Limited access to healthcare, particularly in marginalized areas, paired with geographic barriers to healthcare facilities, further delays timely testing and diagnosis, exacerbating the disease burden [8,9].

The province of Santa Fe, in northeastern Argentina, provides suitable conditions for the proliferation of *Aedes* mosquitoes, including heavy rainfall, flooding, heat waves, and unplanned urban expansion in informal settlements and urban slums [1,2,5]. Santa Fe has experienced dengue outbreaks in 2009, 2016, 2019, 2020, and 2024 [5,6,10]. In 2020 the province reported 4670 confirmed cases, with an incidence rate of 81.6 per 10,000 inhabitants (95% CI: 79.3–84.0), peaking in the epidemiological week 17 [6,10,11]. This outbreak overlapped with the onset of the COVID-19 pandemic in Argentina, presenting a dual challenge for health services [12]. Although several studies have examined dengue in Argentina from 2009 to 2020 [6,9,12,13,14,15,16], most relied on secondary data, with only one study specifically focused on Santa Fe [6].

Sociodemographic and domiciliary factors play a key role in shaping exposure patterns, health behaviors, and environmental conditions that favor DENV transmission [17,18,19,20,21]. Individuals engaged in outdoor occupations, those with limited educational attainment, and children may face heightened risk due to inadequate knowledge or preventive practices [19,20]. In addition, poor housing, irregular garbage collection, lack of access to piped water, presence of uncovered water storage containers, and abandoned properties create optimal breeding grounds for *Aedes* mosquitoes [17,18,22,23].

Knowledge, attitudes, and practices (KAPs) surveys are valuable epidemiological tools for identifying gaps in public awareness, misconceptions about the disease, and inadequate preventive practices, such as improper use of mosquito repellents or insufficient elimination of breeding sites [20,24]. However, despite the recurrent dengue outbreaks in Santa Fe, there is still limited evidence on community KAPs, particularly in socio-environmentally vulnerable peripheral neighborhoods, and on how these factors relate to actual DENV exposure at the household level. When integrated with serological data, KAPs surveys provide a comprehensive understanding of the relationship between community-level awareness and actual infection rates [20,24]. Our study aims to identify KAPs, sociodemographic factors, and environmental factors of the peridomicile related to dengue transmission in three peripheral neighborhoods of Santa Fe, Argentina and examine how these factors are associated with the presence of antibodies against DENV in unreported patients.

## 2. Materials and Methods

### 2.1. Study Setting

**Figure 1:**
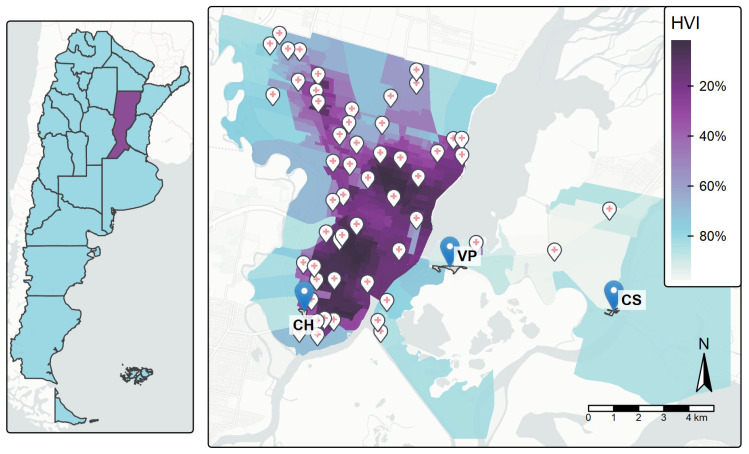
Map of Study Area. The left panel shows the location of the Santa Fe province in Argentina. The right panel shows the spatial distribution of the Health Vulnerability Index (HVI) and the location of healthcare facilities (primary care centers and hospitals) in Santa Fe City; blue pins indicate the locations of the sampled neighborhoods. Spatial layers were obtained from the geoAr R package (Version 1.2.2), OpenStreetMaps^®^ contributors and Rosati et al. (2020) [33], available at: https://mapa.poblaciones.org/map/ (accessed on 19 June 2026).

The city of Santa Fe (31°38′ S 60°42′ W), capital of the province of Santa Fe in Argentina, is surrounded by rivers on the eastern, western, and southern sides, with over 70% of its total surface consisting of rivers, lagoons, and wetlands (Figure 1). Santa Fe experiences a humid subtropical climate, with average annual temperatures ranging between 18–20 °C and annual precipitation averaging 1000–1200 mm [25].

**Figure 2:**
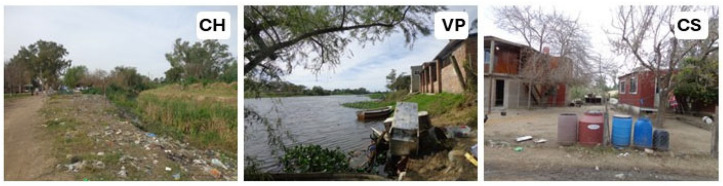
Panel of pictures showing some of the characteristics of the neighborhoods that participated in the study: (CH) Chalet, (VP) Vuelta del Paraguayo, and (CS) Colastiné Sur.

Within the city, socioeconomic conditions are marked by significant disparities [26]. There are 60 marginalized neighborhoods, primarily concentrated in the western part of the city and along the riverside areas [26,27]. Three of these neighborhoods—Chalet (CH), Colastiné Sur (CS), and Vuelta del Paraguayo (VP)—were selected for this study (Figure 1). These neighborhoods present high levels of sanitary vulnerability (Figure 2), with infrastructure deficiencies including unpaved or semi-paved roads, irregular garbage collection, lack of sewage systems, absence of gas networks, and, in the case of CS, lack of access to piped water ((Figure 2); [26]). Additionally, the proximity to public hospitals and healthcare units varies among the selected neighborhoods (Figure 1).

### 2.2. Study Design

Between December 2019 and late March 2020, we conducted a cross-sectional survey targeting residents in the three selected neighborhoods (CS, CS and VP). By selecting these diverse settings (Figure 2), we aimed to explore how KAPs, sociodemographic, and environmental factors might be associated with DENV seropositivity.

Sample size estimation was based on the number of households in the census tracts corresponding to each sampled neighborhood, as reported in the 2010 National Census [28]. The estimation assumed an expected DENV antibody seroprevalence of 50% (due to the lack of previous data), a confidence level of 95%, an absolute precision of 10%, and populations of 277 households for CH, 314 for CS, and 99 for VP. The calculated sample sizes were 72 households for CH, 74 for CS, and 49 for VP. Households were selected randomly within each neighborhood.

### 2.3. Participants

Eligible participants were required to be at least 18 years old and reside in the neighborhood for at least one year. Exclusions from the study were individuals who do not permanently reside in the neighborhood, declined to provide a serum sample for dengue testing, or were ineligible for venipuncture. In order to prevent pseudoreplication, we only sampled one person per household.

### 2.4. Data Collection Tool

The survey instrument consisted of an interviewer-administered questionnaire that covered three modules addressing sociodemographic factors, KAPs regarding dengue, and environmental characteristics of the peridomiciliary area. Participants provided sociodemographic information, including gender (male, female, other), age (in years), highest level of education attained (none, incomplete primary school, complete primary school, incomplete high school, complete high school, incomplete tertiary/university, complete tertiary/university), and occupation (student, homemaker, retired/pensioner, unemployed, underemployed, self-employed, public employee, private employee).

Participants were also asked yes/no, multiple-choice, and open-ended questions regarding their KAPs on dengue. Knowledge questions included yes/no questions on whether participants were aware of dengue, if they knew someone who had had dengue, if it was someone from the neighborhood, if it was someone from the household, and multiple-choice questions assessing knowledge of dengue symptoms and modes of transmission. Attitudes about dengue were also inquired through questions asking participants to compare dengue to leptospirosis (a zoonotic disease endemic in the region), specifically addressing which disease they considered most prevalent, which posed a higher risk of infection, which elicited the greatest fear of contagion, and which received the most publicity. Participants were allowed to select “dengue”, “leptospirosis”, “both”, “neither”, or “not sure”. Additionally, attitudes questions explored actions taken in response to febrile illness symptoms, perceived inconveniences when seeking medical care, and participants’ perceptions of improvements that had taken place in their neighborhood. Practices questions focused on the storage of water containers in the house (yes/no), accumulation of environmental water for cleaning or gardening (yes/no), and garbage disposal methods (garbage collector, incineration, leaving it in the backyard, other/s).

The questionnaire also included yes/no questions on rainwater accumulation on the peridomicile and presence of nearby dump sites, time since the last flooding event (open-ended), and categorized questions about areas reached by flood water (street, front or backyard, inside the house), time taken for the water to recede (hours, days, weeks), and drinking water source (water supply network, pump/well, water truck, other). The research team also conducted direct observations of the peridomiciliary area of each participant, collecting information on street type (paved, semi-paved, dirt, sand), proximity to roadside channels and ditches, dump yards, vacant lots, and water bodies, as well as house floor permeability (permeable, not permeable) and roof permeability (permeable, not permeable).

After answering the questionnaire, the participants received an informative flyer on dengue symptoms, transmission, and prevention. The flyer was explained to the participants, with emphasis on specific aspects based on their responses to the questionnaire.

### 2.5. Data Processing

Age groups of the participants were categorized based on the age quartiles of the sample: 18–31 years, 32–43 years, 44–58 years, 59+ years. Education levels were recategorized into three groups based on the response frequency, as follows: illiterate/incomplete primary school, primary school/incomplete high school, high school and higher. Occupations were re-categorized into four groups, as follows: homemaker/student, retired/pensioner, unemployed/underemployed, employed. Street type was collapsed into three categories, as follows: paved or semi-paved road, dirt road, or sand. The distances to water bodies, roadside channels and ditches, and dump yards were recorded as continuous variables and later re-categorized into three categories, as follows: <25 m, 25–50 m and >50 m.

We created a variable to indicate flooding within 50 m of the house in the past 30 days (yes, no), based on responses regarding the time since the last flooding event. Another variable documented whether participants stored rainwater, derived from the response to a question on the water source for gardening and cleaning. Garbage disposal methods were categorized into four yes/no variables, as follows: garbage truck, burning, accumulation in the backyard, and disposal into the river. A variable indicating whether participants had a previous dengue infection was created, using information from the comments section of the question asking if they knew someone who had been infected with dengue.

Computation of knowledge score was based on four items of the questionnaire each, with a possible range of 0–5 points. The attitudes score was derived from seven items, allowing for a possible range of 0–7 points, while the practices score was calculated based on five items, with a possible range of 0–5 points. Participants who reported not being aware of dengue received a score of 0, while those who had heard about dengue were awarded one point, with additional points assigned based on their responses to the remaining questions. Participants received one point if they reported knowing someone who had had the infection. Based on their response to the question about dengue symptoms, participants were awarded one point if they mentioned fever, and were awarded two points if they mentioned fever along with any of the following symptoms: headache, muscle pain, retro-orbital pain, rash, nausea and vomiting, diarrhea, or hemorrhagic manifestations [7]. Similarly, participants were considered to have knowledge about the modes of transmission and awarded one point if they mentioned mosquito bites, keeping water containers inside or outside the house, or traveling to endemic areas [7].

For the attitudes score, participants were awarded one point if they reported seeking medical care in case of symptoms of febrile illness and zero points if they self-administered medicines or took no action. In addition, participants were awarded one point each if they selected “dengue” or “equally” in the questions about the perceived risk of dengue compared to leptospirosis; another point was awarded if they identified dengue as an important problem in their neighborhood, and one more point if they reported any favorable change in their neighborhood related to dengue prevention.

The practices score was calculated based on each favorable practice, including not storing water containers in the domicile/peridomicile; not using water from the river or lagoon for cleaning, gardening, or consumption; not storing rainwater; not accumulating garbage in the backyard; and disposing garbage for collection by garbage truck.

### 2.6. Laboratory Analysis

Laboratory technicians collected approximately 5 mL of blood from each participant using sterile needles and tubes with coagulation activator and separator gel BD Vacutainer (Becton Dickinson^®^, Franklin Lakes, NJ, USA). After at least 15 min at room temperature, samples were transported to the laboratory, refrigerated at 4–8 °C, centrifuged at 3500 rpm for 10 min, stored at 2–8 °C, and processed within 5 days. Biosafety standards were strictly followed.

The samples were analyzed using an Enzyme-linked immunosorbent assay (ELISA) to detect DENV-specific Immunoglobulin G following the manufacturer’s instructions (Dengue Virus IgG DxSelect, Focus Diagnostics, Cypress, CA, USA), using a Micropar Washer (Micropar I & I^®^, Rosario, Argentina) and a Mindray MR-96A reader (Mindray^®^, Shenzhen, China). The cut-off was calculated for each batch, with the kit’s specificity at 0.93 (95% CI: 0.90–0.96) and sensitivity at 0.96 (95% CI: 0.89–0.99). Results were provided to participants as printed reports, along with a flyer on preventive measures and in-person explanations.

### 2.7. Statistical Analysis

Data were entered into Microsoft Access^®^ and analyzed using R software [29]. We used the package *epiR* [30] to estimate sample sizes per neighborhood, raw seropositivity rates, and adjusted seropositivity rates and their 95% confidence intervals (95% CI).

Frequencies of sociodemographic and household characteristics, as well as KAPs, were compared across neighborhoods using Pearson’s Chi-squared test or Fisher’s exact test for categorical variables, and the Kruskal–Wallis rank-sum test for continuous variables using the package *gtsummary* [31]. The same methods were applied to assess differences in adjusted seroprevalence by neighborhood. Associations between knowledge, attitudes, and practices scores were analyzed using Kendall rank correlation tests.

We fit univariate logistic regression models to examine associations between raw seropositivity and sociodemographic characteristics, environmental factors, and KAPs. Variables with a *p*-value below 0.10 in the univariate models were selected as explanatory variables for multivariate logistic regression models. Two candidate saturated models were compared by their Akaike Information Criteria (AIC) and Bayesian Information Criteria (BIC) using the package performance [32] as follows: (i) study site as random intercept and (ii) study site as a fixed effect. We did not fit models with interaction terms to avoid overfitting of the data and model convergence problems. Non-significant variables were removed via manual step-backwards selection, with inferences drawn from the final model and interpreted using odds ratios (OR) and 95% confidence intervals (95% CI).

## 3. Results

### 3.1. Overview of Study Participants

A total of 272 residents from the three selected neighborhoods were initially considered eligible for the survey. Of these, 81 (29.8%) were excluded for not providing a blood sample; the main reason was the fear of blood extraction. Three additional participants (0.01%) were excluded because, although they were interviewed outside of their homes in public spaces, they resided in households that had already been represented by a previously surveyed participant. After these exclusions, the final sample consisted of 188 participants, 69 (36.7%) from CH, 80 (42.6%) from CS, and 39 (20.7%) from VP. The target sample size was achieved for CS (80 participants enrolled; target, 74 participants). However, participant recruitment was interrupted by the onset of the COVID-19 pandemic before the planned sample sizes for CH and VP could be achieved, which consisted of 72 and 49, respectively.

Of the 188 study participants, 59.6% were female and 40.4% were male, with no significant gender differences across neighborhoods (*p* = 0.807, Table S1). The median age was 45 years (IQR: 32–57 years), but participants from VP were significantly younger (37 years, IQR: 27–44 years) compared to the other neighborhoods (*p* = 0.008, Table S1). The highest level of education attained showed significant variation among neighborhoods (*p* = 0.035), with CH having a notably higher proportion of participants who completed high school (50.7%) compared to CS and VP (Table S1). Regarding occupation, 43.5% of participants were employed, 21.5% were homemakers or students, 20.2% were retired or pensioners, and 14.5% were unemployed or underemployed, with no significant differences between neighborhoods (*p* = 0.176, Table S1).

### 3.2. Environmental Characteristics

The CH site was significantly more urbanized than VP and CS (Figure 2), as reflected by better infrastructure, including a higher proportion of paved or semi-paved roads (84.1%), piped water access (97.1%), greater distance to vacant lots (73.9%), and roadside channels/ditches (72.5%, Table 1). Additionally, CH reported less frequent occurrences of flooding (50.7%), and a higher prevalence of houses with impermeable roofs (94.0%, Table 1). Furthermore, this site was characterized by longer distances from houses to water bodies compared to the other two sites (97.1%, Table 1).

Conversely, the houses in site VP presented significantly higher frequencies of permeable roofs (23.7%), and proximity to roadside channels and ditches (35.9%), dump yards, vacant lots, and water bodies compared to CH and CS (56.4% each, Table 1). All participants from CS reported receiving drinking water delivered by trucks to tanks located next to house fences (Figure 2) or using alternative sources, such as springs, wells, or bottled water, and 56.3% experienced flooding events in the past 30 days (Table 1). No significant differences among neighborhoods were observed in terms of the permeability of house floors, the places reached by flood water, and the time taken for the water to recede (Table 1).

### 3.3. Knowledge, Attitudes, and Practices (KAPs)

Among the 188 participants, 185 (98.4%) reported being aware of dengue, while only three participants from site VP (1.6%) stated they had never heard of it (Table S2). A total of 47 participants from CH (68.1%), 29 (36.3%) from CS, and 3 (8.3%) from VP reported knowing someone who had had dengue, with significant differences among neighborhoods (*p* < 0.001, Table S2). Of those who knew someone with dengue, 68 participants (36.8%) reported knowing someone from their neighborhood, while 14 (7.6%) knew someone from their household, and four participants (2.1%) had had dengue themselves (Table S2). Significant differences were observed between neighborhoods regarding the knowledge of someone who had had dengue (Table S2).

**Figure 3:**
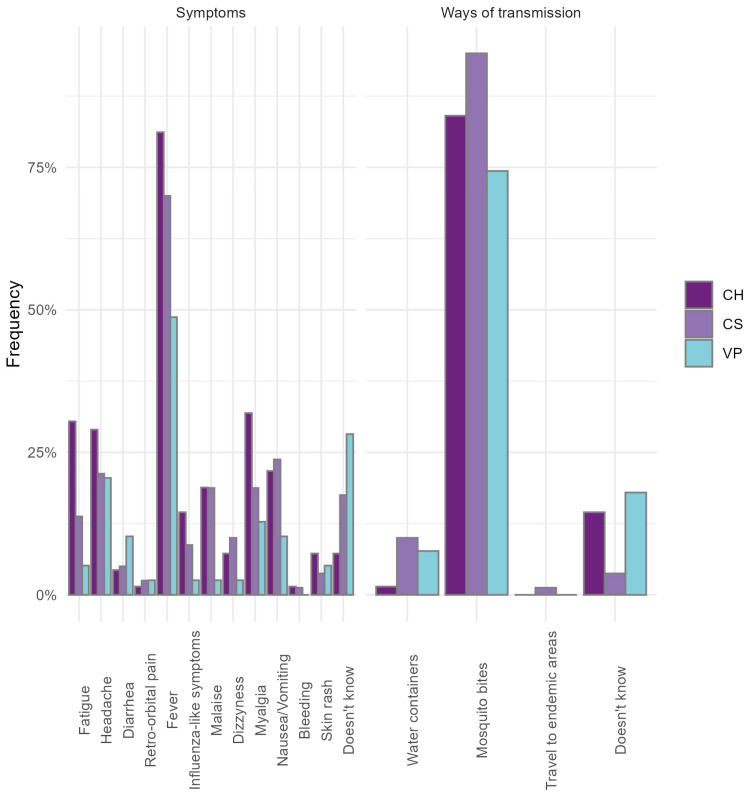
Knowledge about dengue among participants of the survey, stratified by neighborhood (*n* = 181). (**Left**): Dengue symptoms reported; (**Right**): Modes of transmission identified.

Over 45% of participants (45.4%) were able to identify fever along with other symptoms of dengue, 25.4% identified only fever as a symptom of dengue, and 29.2% were unable to identify any symptom of dengue, with significant differences among neighborhoods (*p* = 0.048). The most frequently identified symptoms included fever (70.8%), headache (24.3%), myalgia (22.7%), and nausea/vomiting (20.5%, Figure 3 and Table S2). Significant differences were observed among neighborhoods in the identification of fever as a symptom of dengue (*p* = 0.010, Figure 3 and Table S2). Additionally, 95 participants (50.5%) mentioned non-specific symptoms such as fatigue, malaise, dizziness, and influenza-like symptoms (Figure 3 and Table S2).

Regarding knowledge of dengue transmission, 20 participants (10.8%) were unable to mention any way of transmission, 163 (88.1%) mentioned mosquito bites, 12 (6.5%) mentioned storage of water containers, and only one participant (0.5%) identified traveling to endemic areas as a mode of transmission (Figure 3 and Table S2).

**Figure 4:**
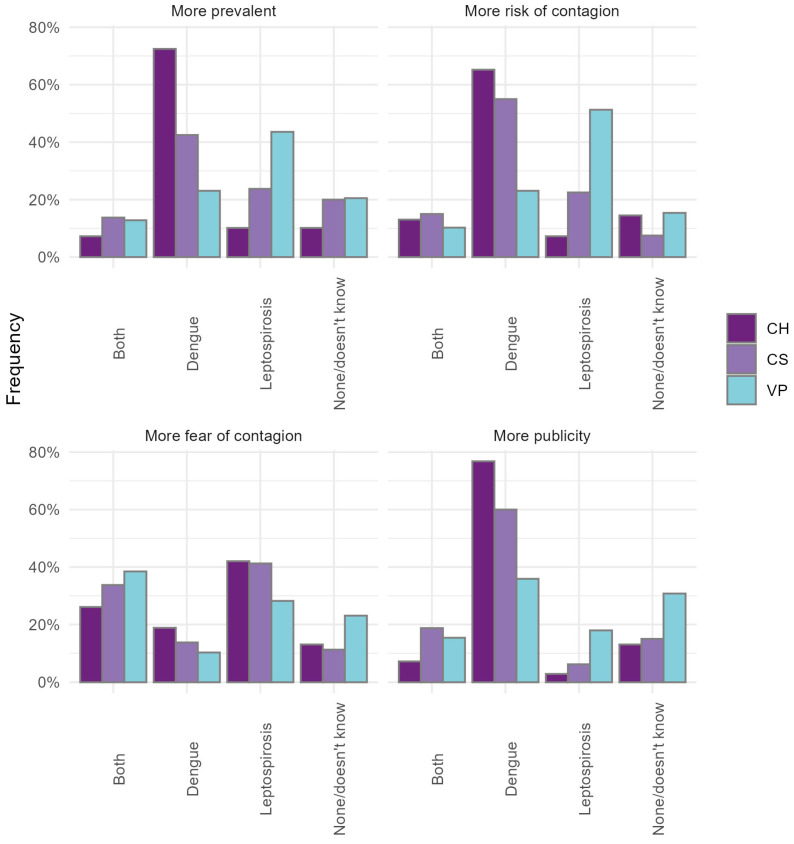
Attitudes towards dengue when compared to leptospirosis among participants of the survey, stratified by neighborhood (*n* = 181).

When asked about their perceptions of the risk of dengue compared to leptospirosis, 93 participants (50.3%) considered dengue to be more prevalent than leptospirosis, 98 (53.0%) believed dengue posed a higher risk of infection, 28 (15.1%) expressed a greater fear of dengue contagion, and 115 (62.2%) indicated that dengue received more publicity (Figure 4 and Table S3). The majority of the participants from site VP considered that they were more exposed to leptospirosis (55.6%, *p* < 0.001), that leptospirosis is more prevalent than dengue (47.2%, *p* < 0.001), and that neither of two receive enough publicity (25%, *p =* 0.001, Figure 4 and Table S3).

The majority of the participants (69.1%) reported experiencing symptoms of febrile illness in the six months prior to the survey (Table S3). The most frequently reported symptoms were headache (40.4%), myalgia (37.8%), and malaise (30.3%), with no significant differences among study sites (*p* > 0.05, Table S3). Of the 120 participants that experienced symptoms, 48.4% sought medical care, 25.4% self-medicated, 24.6% took no action, 1.6% reported taking a different action, and 3.1% did not respond to the question (Table S3). Among those who sought medical care, 15 (25.4%) reported experiencing difficulties. No significant differences were observed among neighborhoods regarding any of the questions related to attitudes towards symptoms of febrile illness (*p* > 0.05).

Sixteen participants (8.5%) were unable to identify any relevant problems in their neighborhood. Among the 172 participants who identified at least one problem, only 4.3% mentioned dengue as a significant issue in their neighborhood (Table S3). Participants from site CH reported the highest percentage of perceived neighborhood improvements (73.9%), which was significantly higher than the frequencies reported for CS and VP (*p* < 0.001, Table S3). However, none of the participants reported any improvements in their neighborhood related to dengue prevention.

Among the study participants, 46.3% reported not storing water containers inside the domicile or in the peridomiciliary area. This behavior was significantly less frequent among participants from site CS (*p* = 0.002, Table S4). The majority of participants reported not using water from rivers or lagoons for consumption, cleaning, or gardening (88.8%), not storing rainwater for cleaning or gardening (92.6%), disposing of garbage in a garbage truck (71.3%), not storing garbage in the peridomiciliary area (88.8%), not burning garbage (79.3%), and not throwing garbage into water bodies (98.4%, Table S4). However, participants from site CS reported significantly lower frequencies of not storing water containers (32.5%, *p* = 0.002) and not collecting rainwater (83.8%, *p* = 0.001) compared to CH and VP (Table S4). Additionally, participants from VP reported significantly lower frequencies of using the garbage truck (17.9%,), not burning garbage (41.0%), and not storing garbage in the peridomiciliary area (69.2%) compared to the other two sites (Table S4).

The knowledge score had a median of 4 points (IQR: 2.5–4) and was significantly lower among participants from site VP (*p* < 0.001, Table S5). The attitudes score had a median of 2 points (IQR: 1–3) and was significantly lower in site VP (*p* < 0.001, Table S5). Similarly, the practices score had a median of 3 points (IQR: 2–3) and was significantly lower in participants from VP (*p* < 0.001, Table S5). According to Kendall’s rank correlation tests, there was a low correlation between knowledge and attitudes scores (29.6%), knowledge and practices scores (24.2%), and attitudes and practices scores (28.8%, Figure S1).

### 3.4. Seropositivity to DENV

Thirty-one out of 188 participants tested positive for IgG antibodies against the dengue virus, resulting in an apparent seropositivity rate of 16.5% (95% CI: 11.9–22.5%, Table S6). After adjusting for the diagnostic test’s sensitivity (96%) and specificity (93%), the adjusted seropositivity rate was 10.7% (95% CI: 5.5–17.4%, Table S6). By neighborhood, the adjusted seropositivity rates were 19.8% in site CH (95% CI: 10.1–32.5%), 9.4% in VP (95% CI: 0.3–25.5%), and 3.4% in CS (95% CI: −2.1–12.9%, Table S6). No significant differences were observed among neighborhoods in either the raw (*p* = 0.2) or adjusted seropositivity rates (*p* = 0.051). Of the seropositive individuals, an estimated 19 (95% CI: 12–28) are likely true positives, while 12 (95% CI: 6–19) are expected to be false positives (Table S6).

A total of 174 participants (92.6%) with complete data on sociodemographic, household, and KAPs variables were included in the analysis. Univariate logistic regression models were fitted to assess associations between raw seropositivity and each of the variables of interest, including gender, age, age group, education level, occupation, type of street, source of drinking water, proximity to roadside channels/ditches, dumpsites, vacant lots, or water bodies, as well as house floor and roof impermeability, flooding or rain-induced flooding (both historically and in the past 30 days), knowing someone who had dengue, awareness of dengue symptoms and modes of transmission, symptoms of febrile illness in the past six months, accumulation of water storage containers, garbage disposal in garbage trucks, and KAPs scores. Statistically significant associations were identified for the type of street (*p* = 0.009), proximity to vacant lots (*p* < 0.001), source of drinking water (*p* = 0.021), storage of water containers (*p* = 0.024), and the presence of febrile illness symptoms in the past six months (*p* = 0.031, Table 2).

Among the two candidate multivariate models, the model with the study site as a random intercept demonstrated the best fit, with an AIC of 148.1 and a BIC of 173.4. This outperformed the model with the study site as a fixed effect (AIC: 149.3, BIC: 177.7). Following a manual backward stepwise selection process, the final model retained only proximity to vacant lots as an explanatory variable. In this model, the variance component for the random effect was nearly negligible (0.005), and living more than 50 m from a vacant lot was associated with about a sixfold increase in the odds of being seropositive for dengue (OR: 5.55, 95% CI: 1.95–15.8). This model explained 18.3% of the observed variability (marginal R^2^: 18.3%, conditional R^2^: 18.4%).

## 4. Discussion

This study is the first to assess dengue seropositivity rates and associated factors in Santa Fe, Argentina, a city with a recent history of DENV outbreaks. Unlike previous studies in the region that relied on reported cases to the Ministry of Health [5,9,11,12,13,14], our study incorporated active serological screening and in situ epidemiological data collection, allowing the identification of previous infections that had not been captured by the routine surveillance system.

Most seropositive individuals identified in this study had not been previously diagnosed or reported as dengue cases, highlighting substantial hidden transmission in the community. Evidence suggests that individuals with asymptomatic or inapparent infections, under certain conditions may be more infectious to mosquitoes than individuals with clinically apparent infections, increasing the risk of outbreak spread within and beyond the city [34]. Furthermore, Vazquez-Prokopec et al. [35] reported that most superspreading units contributing to dengue transmission corresponded to inapparent infections. Additionally, secondary DENV infections with a different serotype, even if the primary infection was asymptomatic or oligosymptomatic, increase the risk of severe dengue hemorrhagic fever [36]. In the Province of Santa Fe, the Ministry of Health prioritizes those with prior documented infections for the vaccination campaigns. However, this prioritization is based solely on medical records. Asymptomatic or unreported cases should also be prioritized in these campaigns due to their increased risk of developing severe illness. The observed adjusted seropositivity (10.7%) indicates unrecognized dengue circulation, consistent with serological findings from other endemic regions in Latin America and Southeast Asia [37,38,39], and reinforcing the international relevance of hidden dengue transmission beyond tropical hyperendemic regions [34,35]. Several mechanisms may explain this under-detection, as follows: a substantial proportion of dengue infections are asymptomatic or oligosymptomatic, symptomatic individuals may not seek medical care due to access barriers or low risk perception, clinical algorithms may fail to identify dengue among other febrile syndromes, and confirmed or suspected cases may not be fully entered into surveillance systems [34,35]. Together, these factors suggest that the true burden of dengue transmission may be considerably underestimated in urban peripheral settings.

Consequently, this study highlights the importance of population screening for estimating dengue seroprevalence in areas with favorable conditions, providing essential data for planning prevention and control strategies and allocating healthcare resources.

The observed seroprevalence was similar to estimates found in blood donors in the Central Region of Argentina, including Santa Fe (12.9%) [37], but lower than in Posadas (20.5%), a city with a longer history of dengue outbreaks [16]. This difference may be due to the persistence of detectable DENV antibodies throughout life, and the greater frequency of outbreaks in Posadas which leads to repeated exposures over time. Our study included individuals over 18 years old, and we did not find significant differences in seropositivity by age group. In contrast, studies in hyperendemic areas have linked age with higher seropositivity due to prolonged exposure and lifelong antibody persistence [37,38]. In Santa Fe, where severe outbreaks started in 2016 [6], all age groups have likely been exposed to the recent outbreaks, which may explain the lack of age variation in our findings, similar to what was observed in Buenos Aires [21].

A central contribution of this study is the combined use of serological data and KAPs surveys. While most participants were aware of dengue as a disease, knowledge was often incomplete and operationally insufficient. Recognition of symptoms and transmission mechanisms did not necessarily translate into protective practices, particularly regarding water storage and waste management. This gap between awareness and effective prevention is epidemiologically relevant because it reveals that knowledge alone is not enough to reduce infection risk. These findings are consistent with previous studies of knowledge, attitudes, and practices (KAPs) and seropositivity to other vector-borne and zoonotic infections, or SARS-COV 2 [20,24,40,41], and point to the need for context-sensitive public health interventions that transcend awareness campaigns and instead address the structural determinants of vulnerability, particularly in informal settlements and flood-prone areas [42,43].

Structural constraints, including irregular water supply, deficient sanitation, flooding, and precarious housing conditions, can limit the adoption of protective behaviors even when people are informed. Integrating serology with KAPs data therefore allows a more complete understanding of the mismatch between perceived risk, actual exposure, and feasible preventive practices.

The main environmental characteristic associated with seropositivity was the presence of vacant lots, a finding that reflects the complex and sometimes contrasting dynamics of dengue transmission. On the one hand, vacant lots may favor the proliferation of *Aedes* mosquitoes by providing unmanaged breeding sites and increasing human exposure to vectors [17,22,23]. On the other hand, lower housing density in these areas may reduce human–vector–human contact and, consequently, the probability of DENV transmission [42]. Although environmental factors such as urban density and peri-domiciliary conditions have been associated with dengue dynamics, our findings suggest that dengue transmission in Santa Fe is better understood as the result of complex socio-environmental vulnerability rather than isolated environmental characteristics. This interpretation is consistent with growing international evidence indicating that dengue risk arises from the interaction between urbanization processes, social inequality, climate variability, and vector ecology [5,14,16,21,42,43].

This study should be interpreted in light of several methodological considerations. Although participant recruitment was interrupted by the onset of the COVID-19 pandemic, preventing us from reaching the planned sample sizes in two of the three study neighborhoods, we were able to enroll most of the target sample. In addition, the multivariable analyses were restricted to participants with complete data, further reducing the effective sample size and limiting our ability to adjust for additional potential confounders. These factors likely contributed to the relatively wide confidence intervals observed for some estimates, which should therefore be interpreted as evidence of association rather than precise measures of effect size. Despite these limitations, as well as possible participation bias and diagnostic uncertainty inherent to ELISA-based serology [44], this study provides valuable evidence on hidden dengue transmission in an emerging temperate endemic setting. Overall, our findings reinforce that dengue epidemiology in southern South America is increasingly shaped by underrecognized infections, urban vulnerability, and surveillance gaps. Strengthening integrated surveillance systems, including serological screening, community engagement, and context-sensitive public health interventions, will be essential to improve early detection, reduce transmission, and mitigate the growing dengue burden in Santa Fe and comparable urban settings worldwide.

Overall, this study reinforces that dengue epidemiology in temperate South America is shaped by asymptomatic transmission, fine-scale spatial heterogeneity, and socio-environmental inequities [20,21,35,42]. Strengthening community-based surveillance and integrating health, environmental, and urban planning policies will be crucial to enhance early detection, mitigate transmission risk, and reduce the long-term burden of dengue in Santa Fe and similar urban-periurban systems.

## Figures and Tables

**Table 1:** Descriptive statistics of peridomiciliary environmental characteristics of survey participants, stratified by neighborhood, Santa Fe, Argentina (2019–2020). Statistically significant differences are indicated in bold.

Characteristic	Overall N = 188	CH N = 69	CS N = 80	VP N = 39	*p*-Value ^1^
**Street type**					**<0.001**
Paved/semi-paved	59 (31.9%)	58 (84.1%)	1 (1.3%)	0 (0.0%)	
Dirt	27 (14.6%)	6 (8.7%)	5 (6.3%)	16 (43.2%)	
Sand	99 (53.5%)	5 (7.2%)	73 (92.4%)	21 (56.8%)	
**Source of drinking water**					**<0.001**
Water pipes	106 (56.7%)	67 (97.1%)	0 (0.0%)	39 (100.0%)	
Tank truck/other	81 (43.3%)	2 (2.9%)	79 (100.0%)	0 (0.0%)	
**Proximity to roadside channels/ditches**					0.641
Over 50 m	129 (68.6%)	50 (72.5%)	54 (67.5%)	25 (64.1%)	
Less than 50 m	59 (31.4%)	19 (27.5%)	26 (32.5%)	14 (35.9%)	
**Proximity to dump yards**					0.079
Less than 50 m	96 (51.1%)	34 (49.3%)	36 (45.0%)	26 (66.7%)	
Over 50 m	92 (48.9%)	35 (50.7%)	44 (55.0%)	13 (33.3%)	
**Proximity to vacant lots**					**<0.001**
Less than 50 m	87 (46.3%)	18 (26.1%)	46 (57.5%)	23 (59.0%)	
Over 50 m	101 (53.7%)	51 (73.9%)	34 (42.5%)	16 (41.0%)	
**Proximity to water bodies**					**<0.001**
Over 50 m	117 (62.2%)	67 (97.1%)	39 (48.8%)	11 (28.2%)	
Less than 50 m	71 (37.8%)	2 (2.9%)	41 (51.3%)	28 (71.8%)	
**Permeable house floor**	171 (94.0%)	64 (95.5%)	73 (94.8%)	34 (89.5%)	0.439
**Impermeable house roof**	163 (89.6%)	63 (94.0%)	71 (92.2%)	29 (76.3%)	**0.018**
**History of floodings**	139 (73.9%)	35 (50.7%)	68 (85.0%)	36 (92.3%)	**<0.001**
**Floodings in the past 30 days**	65 (34.6%)	9 (13.0%)	45 (56.3%)	11 (28.2%)	**<0.001**
**Area reached for the flood (past 30 days)**					**0.036**
House	36 (24.0%)	8 (17.4%)	13 (18.8%)	15 (42.9%)	
Street	53 (35.3%)	19 (41.3%)	23 (33.3%)	11 (31.4%)	
Front or backyard	61 (40.7%)	19 (41.3%)	33 (47.8%)	9 (25.7%)	
**Time taken for the water to recede (past 30 days)**					0.109
Days	28 (43.1%)	2 (22.2%)	22 (48.9%)	4 (36.4%)	
Hours	27 (41.5%)	7 (77.8%)	14 (31.1%)	6 (54.5%)	
Weeks	10 (15.4%)	0 (0.0%)	9 (20.0%)	1 (9.1%)	

^1^ Pearson’s Chi-squared test; Fisher’s exact test.

**Table 2:** Univariate mixed-effects logistic regression models of factors associated with DENV seropositivity in Santa Fe, Argentina, 2019–2020. Among the 174 participants included in the analysis, 29 were DENV seropositive. Neighborhood was included as a random intercept in all models. Statistically significant associations are shown in bold.

Characteristic	OR	95% CI	*p*-Value
**Sex**			
Female	—	—	
Male	1.46	0.65, 3.28	0.4
**Age (years)**	1.02	1.00, 1.05	0.095
**Age group**			
18–31	—	—	
32–43	1.36	0.35, 5.28	0.7
44–58	2.51	0.72, 8.77	0.15
59+	2.13	0.58, 7.89	0.3
**Education**			
None/incomplete primary school	—	—	
Primary school	0.65	0.18, 2.32	0.5
High school/university	1.12	0.32, 3.92	0.9
**Occupation**			
Homemaker/student	—	—	
Employed	1.44	0.47, 4.40	0.5
Unemployed/underemployed	0.77	0.16, 3.65	0.7
Retired/pensioner	1.23	0.33, 4.54	0.8
**Street type**			
Paved/semi-paved	—	—	
Dirt	1.34	0.46, 3.93	0.6
Sand	0.29	0.11, 0.73	**0.009**
**Roadside channels/ditches**	0.48	0.18, 1.25	0.13
**Proximity to roadside channels/ditches**			
Over 50 m	—	—	
Less than 50 m	0.51	0.19, 1.35	0.2
**Dump yards**	0.65	0.28, 1.48	0.3
**Proximity to dump yards**			
Less than 50 m	—	—	
Over 50 m	1.31	0.58, 2.96	0.5
**Vacant lots**	0.30	0.13, 0.68	**0.004**
**Proximity to vacant lots**			
Less than 50 m	—	—	
Over 50 m	5.55	1.95, 15.8	**0.001**
**Water bodies**	0.74	0.27, 2.00	0.6
**Proximity to water bodies**			
Over 50 m	—	—	
Less than 50 m	0.78	0.28, 2.13	0.6
**Source of drinking water**			
Water pipes	—	—	
Tank truck/other	0.34	0.14, 0.85	**0.021**
**Permeable house floor**	2.10	0.25, 17.3	0.5
**Impermeable house roof**	0.65	0.19, 2.21	0.5
**History of floodings**	1.50	0.54, 4.15	0.4
**Floodings in the past 30 days**	0.94	0.34, 2.57	>0.9
**Knows someone with dengue**	1.67	0.71, 3.92	0.2
**Knows any symptom**	0.94	0.58, 1.53	0.8
**Any way of transmission**	4.84	0.60, 38.7	0.14
**Any febrile illness symptom**	0.42	0.18, 0.95	**0.036**
**Stores water containers**	0.41	0.17, 0.97	**0.043**
**Disposes in the garbage truck**	1.62	0.55, 4.77	0.4
**Knowledge score**	1.14	0.80, 1.62	0.5
**Attitudes score**	1.06	0.76, 1.47	0.7
**Practices score**	1.71	0.80, 3.66	0.2

Abbreviations: OR = Odds-ratio; CI = Confidence Interval.

## Data Availability

Datasets have been anonymized to remove personally identifiable information. The Supplementary Material, datasets, and R scripts used in this manuscript are available at: https://doi.org/10.5281/zenodo.17486804.

## Supplementary Materials

The following supporting information can be downloaded at: https://www.mdpi.com/article/10.3390/epidemiologia7040099/s1, Table S1: Demographic data of the study participants. Table S2: Knowledge about dengue by neighborhood. Table S3: Perceptions of the risk of dengue compared to leptospirosis among participants. Table S4: Preventive practices against dengue among the study participants. Table S5: KAPs scores by neighborhood. Table S6: ELISA positivity and DENV seroprevalence by neighborhood. Figure S1: Kendall’s correlation among KAPs scores.

## Abbreviations

The following abbreviations are used in this manuscript:

DENV	Dengue virus
KAPs	Knowledge-attitudes-practices
IgG	Immunoglobulin G
ELISA	Enzyme Linked Immunosorbent Assay
EID	Emerging Infectious Disease
CH	Chalet
CS	Colastiné Sur
VP	Vuelta del Paraguayo
AIC	Akaike Information Criteria
BIC	Bayesian Information Criteria
