# Supplementary material for: Unrecognized Dengue Transmission in Socially Vulnerable Peri-Urban Neighborhoods of a Temperate Argentine City: Integrating Serology with Knowledge, Attitudes, and Practices
Source: Epidemiologia (Basel). 2026 Jul 13;7(4):99. doi: 10.3390/epidemiologia7040099 (PMC13397892; doi:10.3390/epidemiologia7040099)
Supplement: Supplementary file 1 [file epidemiologia-07-00099-s001.zip › epidemiologia-4331618-supplementary.pdf]

Supplementary material

Table S1. Demographic data of the study participants, Santa Fe, Argentina (2019-2020).

Characteristic	Overall<br>N = 188	CH<br>N = 69	CS<br>N = 80	VP<br>N = 39	p-value[1]
**Sex**					0.807
Female	112 (59.6%)	40 (58.0%)	47 (58.8%)	25 (64.1%)	
Male	76 (40.4%)	29 (42.0%)	33 (41.3%)	14 (35.9%)	
**Education**					**0.035**
None/incomplete primary school	24 (12.9%)	8 (11.9%)	9 (11.3%)	7 (17.9%)	
Primary school	91 (48.9%)	25 (37.3%)	42 (52.5%)	24 (61.5%)	
High school/university	71 (38.2%)	34 (50.7%)	29 (36.3%)	8 (20.5%)	
**Occupation**					0.176
Homemaker/student	40 (21.5%)	11 (16.2%)	15 (18.8%)	14 (36.8%)	
Employed	81 (43.5%)	32 (47.1%)	35 (43.8%)	14 (36.8%)	
Unemployed/underemployed	27 (14.5%)	11 (16.2%)	10 (12.5%)	6 (15.8%)	
Retired/pensioner	38 (20.4%)	14 (20.6%)	20 (25.0%)	4 (10.5%)	

[1]Pearson's Chi-squared test

Table S2. Knowledge about dengue by neighborhood, Santa Fe, Argentina (2019-2020).

Characteristic	**Overall** N = 188	**CH** N = 69	**CS** N = 80	**VP** N = 39	**p-value**[1]
**Is aware of dengue**	185 (98.4%)	69 (100.0%)	80 (100.0%)	36 (92.3%)	**0.008**
**Knows someone with dengue**	79 (42.7%)	47 (68.1%)	29 (36.3%)	3 (8.3%)	**<0.001**
**Someone from neighborhood**	68 (36.8%)	45 (65.2%)	23 (28.8%)	0 (0.0%)	**<0.001**
**Someone from household**	14 (7.6%)	11 (15.9%)	2 (2.5%)	1 (2.8%)	**0.006**
**Had dengue themself**	4 (2.1%)	3 (4.3%)	1 (1.3%)	0 (0.0%)	0.427
Dengue symptoms					
**Fever**	131 (70.8%)	56 (81.2%)	56 (70.0%)	19 (52.8%)	**0.010**
**Headache**	45 (24.3%)	20 (29.0%)	17 (21.3%)	8 (22.2%)	0.519
**Myalgia**	42 (22.7%)	22 (31.9%)	15 (18.8%)	5 (13.9%)	0.060
**Diarrhea**	11 (5.9%)	3 (4.3%)	4 (5.0%)	4 (11.1%)	0.361
**Nausea/vomiting**	38 (20.5%)	15 (21.7%)	19 (23.8%)	4 (11.1%)	0.283
**Malaise**	29 (15.7%)	13 (18.8%)	15 (18.8%)	1 (2.8%)	0.060
**Fatigue**	34 (18.4%)	21 (30.4%)	11 (13.8%)	2 (5.6%)	**0.003**
**Skin rash**	10 (5.4%)	5 (7.2%)	3 (3.8%)	2 (5.6%)	0.640
**Hemorrhage**	2 (1.1%)	1 (1.4%)	1 (1.3%)	0 (0.0%)	>0.999
**Influenza-like symptoms**	18 (9.7%)	10 (14.5%)	7 (8.8%)	1 (2.8%)	0.168
**Dizzyness**	14 (7.6%)	5 (7.2%)	8 (10.0%)	1 (2.8%)	0.478
**Retro-orbital pain**	4 (2.2%)	1 (1.4%)	2 (2.5%)	1 (2.8%)	>0.999
**None**	30 (16.2%)	5 (7.2%)	14 (17.5%)	11 (30.6%)	**0.008**
Ways of transmission					
**Mosquito bites**	163 (88.1%)	58 (84.1%)	76 (95.0%)	29 (80.6%)	**0.027**
**Water containers**	12 (6.5%)	1 (1.4%)	8 (10.0%)	3 (8.3%)	0.069
**Travel to endemic areas**	1 (0.5%)	0 (0.0%)	1 (1.3%)	0 (0.0%)	>0.999
**Doesn't know any way of transmission**	20 (10.8%)	10 (14.5%)	3 (3.8%)	7 (19.4%)	**0.012**

[1]Fisher's exact test; Pearson's Chi-squared test

Table S3: Perceptions of the risk of dengue compared to leptospirosis among participants, Santa Fe, Argentina (2019-2020).

**Characteristic**	**Overall** N = 188	**CH** N = 69	**CS** N = 80	**VP** N = 39	**p-value**[1]
**Most fear of contagion**					0.622
Leptospirosis	73 (39.5%)	29 (42.0%)	33 (41.3%)	11 (30.6%)	
Both	60 (32.4%)	18 (26.1%)	27 (33.8%)	15 (41.7%)	
Dengue	28 (15.1%)	13 (18.8%)	11 (13.8%)	4 (11.1%)	
None/not sure	24 (13.0%)	9 (13.0%)	9 (11.3%)	6 (16.7%)	
**More risk of infection**					
Dengue	98 (53.0%)	45 (65.2%)	44 (55.0%)	9 (25.0%)	
Both	25 (13.5%)	9 (13.0%)	12 (15.0%)	4 (11.1%)	
None/not sure	19 (10.3%)	10 (14.5%)	6 (7.5%)	3 (8.3%)	
Leptospirosis	43 (23.2%)	5 (7.2%)	18 (22.5%)	20 (55.6%)	
**Higher prevalence**					
Dengue	93 (50.3%)	50 (72.5%)	34 (42.5%)	9 (25.0%)	
Both	21 (11.4%)	5 (7.2%)	11 (13.8%)	5 (13.9%)	
Leptospirosis	43 (23.2%)	7 (10.1%)	19 (23.8%)	17 (47.2%)	
None/not sure	28 (15.1%)	7 (10.1%)	16 (20.0%)	5 (13.9%)	
**More publicity**					**0.003**
Both	26 (14.1%)	5 (7.2%)	15 (18.8%)	6 (16.7%)	
Dengue	115 (62.2%)	53 (76.8%)	48 (60.0%)	14 (38.9%)	
Leptospirosis	14 (7.6%)	2 (2.9%)	5 (6.3%)	7 (19.4%)	
None/not sure	30 (16.2%)	9 (13.0%)	12 (15.0%)	9 (25.0%)	
Febrile illness symptoms (6 months)					
**Any febrile illness symptom**	130 (69.1%)	46 (66.7%)	58 (72.5%)	26 (66.7%)	0.693
**Headache**	76 (40.4%)	23 (33.3%)	36 (45.0%)	17 (43.6%)	0.317
**Myalgia**	71 (37.8%)	26 (37.7%)	30 (37.5%)	15 (38.5%)	0.995
**Diarrhea/vomiting**	28 (14.9%)	8 (11.6%)	13 (16.3%)	7 (17.9%)	0.608
**Skin rash**	20 (10.6%)	8 (11.6%)	10 (12.5%)	2 (5.1%)	0.511
**Malaise**	57 (30.3%)	18 (26.1%)	28 (35.0%)	11 (28.2%)	0.473
**Fever**	19 (10.1%)	5 (7.2%)	8 (10.0%)	6 (15.4%)	0.400
**Action taken**					0.306
Sought medical care	61 (48.4%)	23 (52.3%)	30 (52.6%)	8 (32.0%)	
Self-medicated	32 (25.4%)	9 (20.5%)	14 (24.6%)	9 (36.0%)	
Nothing	31 (24.6%)	10 (22.7%)	13 (22.8%)	8 (32.0%)	

**Characteristic**	**Overall** N = 188	**CH** N = 69	**CS** N = 80	**VP** N = 39	**p-value**[1]
Other	2 (1.6%)	2 (4.5%)	0 (0.0%)	0 (0.0%)	
**Issues in the neighborhood**	172 (91.5%)	56 (81.2%)	78 (97.5%)	38 (97.4%)	**<0.001**
**Dengue as an issue in the neighborhood**	8 (4.3%)	3 (4.3%)	3 (3.8%)	2 (5.1%)	0.903
**Neighborhood improvements**	76 (40.4%)	51 (73.9%)	19 (23.8%)	6 (15.4%)	**<0.001**

[1]Fisher's exact test; NA; Pearson's Chi-squared test

Table S4: Preventive practices against dengue among the study participants, Santa Fe, Argentina (2019-2020).

Characteristic	Overall N = 188	CH N = 69	CS N = 80	VP N = 39	p-value[1]
Avoids storage of environmental water					
**Storing water containers**	87 (46.3%)	42 (60.9%)	26 (32.5%)	19 (48.7%)	**0.002**
**Using water from rivers/lagoons**	167 (88.8%)	65 (94.2%)	70 (87.5%)	32 (82.1%)	0.132
**Using rainwater**	174 (92.6%)	68 (98.6%)	67 (83.8%)	39 (100.0%)	**<0.001**
Garbage disposal					
**Disposes in the garbage truck**	134 (71.3%)	64 (92.8%)	63 (78.8%)	7 (17.9%)	**<0.001**
**Avoids burning**	149 (79.3%)	68 (98.6%)	65 (81.3%)	16 (41.0%)	**<0.001**
**Avoids storing in the backyard**	167 (88.8%)	69 (100.0%)	71 (88.8%)	27 (69.2%)	**<0.001**
**Avoids throwing into water bodies**	185 (98.4%)	69 (100.0%)	79 (98.8%)	37 (94.9%)	0.158

[1]Pearson's Chi-squared test; Fisher's exact test

Table S5: KAPs scores by neighborhood, Santa Fe, Argentina (2019-2020).

Characteristic	Overall N = 188	CH N = 69	CS N = 80	VP N = 39	p-value[1]
**Knowledge score**	4.00 (2.50, 4.00)	4.00 (3.00, 5.00)	4.00 (3.00, 4.00)	2.00 (2.00, 4.00)	**<0.001**
**Attitudes score**	2.00 (1.00, 3.00)	3.00 (2.00, 3.00)	2.00 (1.00, 3.00)	1.00 (0.00, 1.00)	**<0.001**
**Practices score**	3.00 (2.00, 3.00)	3.00 (3.00, 3.00)	3.00 (2.00, 3.00)	2.00 (1.00, 2.00)	**<0.001**

[1]Kruskal-Wallis rank sum test

Figure S1: Kendall's correlation among KAPs scores, Santa Fe, Argentina (2019-2020).

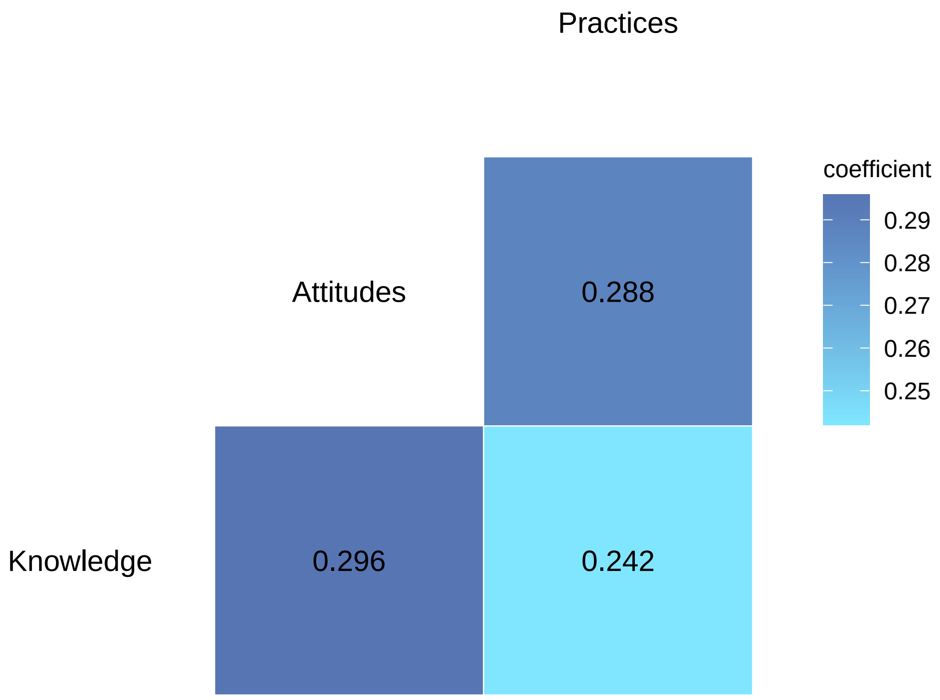

Table S7: ELISA positivity and DENV seroprevalence by neighborhood, Santa Fe, Argentina (2019-2020).

Site	Positives			Seroprevalence	
	Test	True	False	Apparent	True
CH	17.00 (10.00-24.00)	13.00 (7.00-20.00)	4.00 (1.00-8.00)	0.25 (0.16-0.36)	0.20 (0.10-0.33)
CS	8.00 (3.00-14.00)	2.00 (-0.00-6.00)	5.00 (1.00-10.00)	0.10 (0.05-0.19)	0.03 (-0.02-0.13)
VP	6.00 (2.00-11.00)	3.00 (1.00-7.00)	2.00 (-0.00-6.00)	0.15 (0.07-0.30)	0.09 (0.00-0.26)
Total	31.00 (21.00-41.00)	19.00 (12.00-28.00)	12.00 (6.00-19.00)	0.16 (0.12-0.22)	0.11 (0.05-0.17)
